# Randomized trial comparing group size of periodic in-person sessions in a remotely delivered weight loss intervention

**DOI:** 10.1186/s12966-017-0599-3

**Published:** 2017-10-23

**Authors:** Deborah F. Tate, Carmina G. Valle, Melissa M. Crane, Brooke T. Nezami, Carmen D. Samuel-Hodge, Karen E. Hatley, Molly Diamond, Kristen Polzien

**Affiliations:** 1 Department of Health Behavior, Department of Nutrition, The University of North Carolina at Chapel Hill, Chapel Hill, NC 27599-7440 USA; 2 Department of Nutrition, The University of North Carolina at Chapel Hill, Chapel Hill, NC 27599-7294 USA; 3 Department of Preventive Medicine, Rush University Medical Center, 1700 W. Van Buren St., Suite 470, Chicago, IL 60612 USA; 4 Lineberger Comprehensive Cancer Center, The University of North Carolina at Chapel Hill, Chapel Hill, NC 27599-7294 USA; 5 Department of Nutrition, The University of North Carolina at Chapel Hill, Chapel Hill, NC 27599-7426 USA; 6 Lineberger Comprehensive Cancer Center, The University of North Carolina at Chapel Hill, Chapel Hill, NC 27599-7294 USA

**Keywords:** Hybrid internet plus in-person groups, Large group, Behavioral weight loss

## Abstract

**Background:**

Few randomized studies have examined differential effects of group size in behavioral weight control, especially in hybrid programs that include Internet treatment approaches.

**Methods:**

Randomized controlled trial (*n* = 195) comparing a 4 month hybrid internet weight loss program coupled with monthly face to face groups of 100 persons (Large Group, LG; 1 group) or to the same approach with monthly groups of 20 persons (Small Group, SG; 4 groups). Repeated-measures mixed-model analysis with age and race as covariates were used to estimate primary (weight) and secondary outcomes, and to test group differences in change over time.

**Results:**

The sample was 46.3 years old ±10.4, 90.3% female, and 51.9% non-white, with BMI 37.9 ± 8.4 kg/m^2^. Participants in the LG were more likely to return for the 4-month assessment visit than those in the SG (*p* = 0.04). Participants randomized to both the LG and SG conditions experienced significant WL over time (no between group difference: −4.1 kg and −3.7 kg, respectively) and weight loss was positively associated with attendance at monthly meetings and logins to the website. Satisfaction with the program was high and similar in both groups (94.4% reported that they were “satisfied” or “very satisfied”).

**Conclusions:**

Using a hybrid approach of in-person and online weight loss interventions may be an effective way to reach larger and more diverse populations. Delivering the face to face component of the intervention in groups larger than those traditionally delivered (20–25 people) could increase the cost-effectiveness of group-based behavioral weight loss interventions.

**Clinical trials registration number:**

NCT01615471. Registered June 6, 2012. Registered retrospectively.

## Background

The majority of US adults are overweight and obese [1], and studies show that even modest weight loss of approximately 5% of initial body weight can improve health parameters like cholesterol, blood glucose, and blood pressure, and reduce risk for chronic diseases like diabetes and cardiovascular disease [2, 3]. More recent evidence links obesity to progression and recurrence of some cancers [2–4]. Therefore, for the majority of US adults, risk reduction through modest weight loss is a public health priority.

Intensive behavioral lifestyle interventions have been developed and are proven to reduce weight in clinical trials at levels that are beneficial for health [5–8]. The 2014 American Heart Association evidence-based guidelines on the treatment of obesity suggest that lifestyle interventions are most successful when they include face-to-face sessions [6]. Research also suggests that both individual and group sessions are effective delivery formats for face-to-face sessions in behavioral lifestyle interventions [6–8], and that group format may be more effective and cost-effective for delivery of comprehensive treatment [9].

Other than the comparison of individual with group delivery formats, little research has examined the effect of group size on weight loss. The Look Ahead study delivered 3 of 4 sessions per month in group format with an average group size of 11 participants; groups ranged from 6 to 21 participants over the 209 groups delivered across the 15 sites [10]. In an analysis controlling for site differences, group size was not found to be associated with mean weight loss [10]. In the single randomized trial conducted to date on this topic in obesity, Dutton and colleagues examined the effects of group size on engagement and weight loss [5]. Sixty-six participants were randomized to one group of 30 or to one of three smaller groups of 12 members. Behavioral weight loss treatment was delivered with 24 weekly groups (months 1–6) followed by 6 monthly sessions (months 7–12). Weight losses were over 3 kg and 4 kg greater in the smaller groups versus larger group at 6 and 12 months respectively. Participants in the smaller groups completed significantly more self-monitoring records (*p* < .01) than the larger group, and there was a trend for smaller group participants to attend more sessions (*p* = .09), at least partially explaining the differences in weight loss between groups. Thus, the limited available evidence is mixed regarding group size and weight loss outcomes and no data exists on group sizes above 20–25 persons. With dissemination of group based treatment in mind, examination of larger group sizes is needed as the cost of in-person treatment delivery is affected by the number of sessions needed and also the number of individuals who can be treated in each group session.

The cost, logistics and accessibility of comprehensive group behavioral lifestyle interventions have resulted in much research over the past decade on alternative delivery formats and locations for weight loss interventions. One promising alternative has been delivery via technology, including Internet interventions accessed via computer and mobile devices. In general, weight losses of approximately 4–6 kg can be reliably achieved with Internet programs that involve some form of weekly human e-counseling or ongoing feedback from behavioral lifestyle counselors (email, group chat, etc.) [11–16]. Research also suggests the efficacy of weekly professionally led group chat visits as part of an internet program [17, 18] but less is known about periodic human support delivered either in person or online.

The combination of periodic face-to-face visits and internet delivery might be appealing for several reasons: it retains some of the benefits of face-to-face delivery through participant interaction and modeling; it provides opportunity for greater accountability with periodic in-person weigh-ins; and using Internet intervention components has the potential to reduce labor costs in program delivery, allows constant access to ongoing treatment components, and increased convenience for participants. One study examined a hybrid approach delivered predominantly via weekly Internet chat groups (15–20 individuals), coupled with individual sessions once per month with a dietitian. Results suggested no additional benefit from the monthly individual face-to-face sessions, given an effective Internet program that included participant-provider interaction and participant-participant interaction within the online treatment [13]. Another study by Leahey and colleagues [19] added optional weekly group-delivered weight loss sessions to a 3-month online behavioral weight loss program and demonstrated no differences at the end of treatment (5.8% vs. 4.3% loss), but significantly greater weight losses were maintained 1 year after baseline (9 months post-treatment: optional groups 4.5% loss vs. 1.2% for internet only).

Given that individual treatment is more costly to deliver than group treatment [9], and that Internet delivered programs are largely accessed by individuals on their own, we reasoned that an Internet- and mobile-delivered program could be combined with periodic group treatment and that it might promote ongoing engagement with the program when delivered in a community setting. Another reason for offering a hybrid program in a community setting was to increase potential to appeal to a broader audience since treatment was delivered via multiple formats. Since little data existed on group size, we also sought to explore the feasibility of delivering the face-to-face component of the hybrid program in a larger group of 100 compared with more traditional large group sizes of 25. Therefore, the objective of this study was to evaluate the feasibility and preliminary efficacy of a 4-month Internet- and mobile-delivered weight loss program with monthly face-to-face group sessions delivered in a large group format (approximately 100 person group) compared with the Internet and mobile program delivered via a smaller group format (20 person groups).

## Methods

### Recruitment of intervention participants

We recruited and enrolled participants between April and June 2012 in Kannapolis, NC and the surrounding area, beginning the intervention in mid-June. A comprehensive recruitment website was developed to provide details about the study, eligibility, a video of a former research participant (African American female) talking about study participation, general information about participating in a research study and frequently asked questions. Potential participants were recruited through television advertisements, flyers and word of mouth and directed to the recruitment website, which linked to an online eligibility screener. Individuals were eligible if they met the following criteria: overweight and obese (BMI ≥25 kg/m^2^); aged 18–65 years old; living or working within 30 miles of Kannapolis; able to attend 4 monthly Saturday group sessions and 2 assessment visits at the Nutrition Research Institute in Kannapolis; had access to the Internet on at least a weekly basis; and had an email address or were willing to sign up for a free account (e.g., gmail). Participants were excluded if they were unable to read and write English, taking insulin, pregnant during the previous 3 months or planning pregnancy in the following 6 months, participating in another weight loss program, or were taking weight loss medications. Though no upper BMI limit was set, physician consent was required for individuals with BMI > 50, previous history of heart attack or stroke, or current treatment for cancer. To ensure participant safety, The Physical Activity Readiness Questionnaires (PAR-Q) was administered at the baseline assessment visit, and those endorsing any item on the PAR-Q were encouraged to consult their primary care physician prior to making any changes to their exercise routine.

To aid with recruitment into a study where participants could be randomized to come to a large treatment group with 100 people, participants could join the study alone but were encouraged to enroll in the study with up to three friends or family members who also met eligibility criteria. Participants were oriented (video), provided informed consent, and enrolled online. A paper copy of the consent was provided to the participant at the baseline assessment visit. This study was approved by and was in accordance with the ethical standards of the UNC Chapel Hill Institutional Review Board.

### Randomization

Participants in the Lose Now NC intervention trial were randomized to one of 2 intervention groups: a “large group format” (LG) in which monthly sessions would include up to 100 participants, or a “small group format” (SG) in which monthly sessions would include approximately 20 participants. Randomization was stratified on the size of the “friends or family” social group joining together (1 = joining alone, 2, 3, or 4). Eligible participants were randomized following baseline measures, using a computer generated random numbers method by the project coordinator with allocation concealed to participants until randomization was revealed at the initial group session.

### Intervention

A standard behavioral intervention based on the DPP, and adapted for use by our research team in other internet and face-to-face delivered studies [11, 12, 20–23] was adapted for this protocol. Behavioral weight control approaches are founded on teaching self-regulatory and behavioral skills (self-monitoring, goal setting, problem solving, stimulus control, etc.) and providing the support necessary to enable participants to adopt lower calorie diets (e.g., 1500–2000 kcals per day based on starting weight) and moderate physical activity (e.g., walking) to produce energy deficits necessary to produce modest weight losses of 1–2 lbs. per week. Both treatment groups attended four monthly group sessions and had access to an identical Internet program in between sessions. The main difference between the two study groups was the size of the monthly group sessions.

Monthly Treatment Groups: Treatment group sessions were held in a community facility equipped with a large room with chairs, a podium, and audio-visual equipment, which would accommodate 150 people. Smaller rooms with conference style tables and audio-visual equipment were also used. At the beginning of the program, all participants were provided with a “weight loss toolkit” to encourage evidence-based weight loss behaviors and for retention purposes. The kit included a 16 oz. water cup, Meal Measure portion control plate, and a Calorie King calorie counting guide [24]. They also were given a pedometer (PE-330 Step Tri-Axis Pocket Pedometer) at the second group session to correspond with the lifestyle physical activity lesson topic. Group sessions were held once a month on a Saturday and were approximately 90 min in duration. Over the 4 months, the same interventionists co-led the large group and individually led the small groups. Interventionists were from a multi-disciplinary team of dietitians, psychologists, exercise physiologists and health behavior experts and were trained at the Master’s or PhD level. The monthly sessions began with an individual, semi-private weigh-in, followed by a group session. Both groups received the same paper copy of the ‘lesson’ for the session, but delivery of the content followed a slightly different format. In the LG, there were interactive features such as group text polling, cooking and exercise demonstrations, information tables for browsing before and after the session, and audience participation using a microphone. The SG sessions followed traditional behavioral weight control group session protocol with groups sitting in a small group or circle format with a table and allowing for more interaction and discussion between members and with the leader.

Internet and Mobile Program: Between face-to-face sessions, participants were asked to log into a comprehensive Internet program adapted from those tested previously by Tate et al. [11, 12, 22] that provides the self-monitoring tools, tailored automated behavioral feedback, tailored lifestyle content resources (lessons and a problem solving tool to help overcome barriers to diet and exercise change), and social support (e.g., online message board). New lesson content was posted weekly, and included information on making dietary changes, goal setting, exercise barriers, and problem solving. Presentation of the content included text, videos and interactive questionnaires. The expected frequency of Internet use was at least once a week in order to report weight, diet and exercise, and to use the program tools.

Weighing, Diet, and Exercise Recommendations and Self-Monitoring Options: Participants were instructed to weigh daily and to report weight on the study website. At session one, participants were given calorie goals designed to produce 1–2 lb. weight loss per week based on starting weight. They were given three “plans” designed to help them meet their prescribed weight loss calorie goal. The first plan offered was to self-select their diet and use the website diary to monitor all calories consumed and meet the calorie goal. The website diary used the Calorie King database and was fashioned after many commercial online diet self-monitoring tools. The second was to use an external diary (commercial smartphone app, other web-based diary or paper diary), and to enter total daily calories and fat to the website at the end of the week. The third option was to follow a study-provided meal plan at the recommended reduced calorie level and indicate only deviations to the meal plan on the study website diary. No preference was proposed for the three plans, however, the “pros” and “cons” to each were presented at the first session, and participants were encouraged to select the plan that fit best with their lifestyle and preferences for self-monitoring.

Participants also were asked to select a physical activity progression plan that took into account their baseline level of activity and progressed gradually towards an increased goal during the intervention. Plans for baseline “low active” (<60 min/week), “somewhat active” (60–150 min/week), and “highly active” (>150 min/week) levels recommended a progression over the 16-week intervention that increased the weekly minutes to 175, 200, and 250, respectively. Participants were asked to report their exercise minutes on the website. An online exercise scheduler and exercise barrier problem solving tool were provided to promote adherence to the recommendations. The pedometer was provided, after one month, as an additional tool for monitoring total activity and reporting steps on the study website was optional.

### Measures

Assessments occurred at baseline and 4 months in person and using online questionnaires. Basic demographic information, including age, sex, race, education level, and income, was assessed at baseline only. Body weight was measured in lightweight street clothes, without shoes, on a calibrated digital scale (Tanita BWB 800) by blinded assessors. Height was measured at baseline using a portable stadiometer (Seca). Resting blood pressure was measured in seated position using a GE Dinamap ProCare 100 after 5 min rest; the average of two measures was used [25]. If there was a discrepancy of >10 mmHg systolic or >6 mmHg diastolic, a third measure was taken and the average of the three measures was used. Non-fasting blood samples were collected by fingerstick according to standard protocol. Hemoglobin A1C (HgA1C) was analyzed using an Alere Afinion (Orlando, Florida) point of care analyzer. Blood lipids (triglycerides, total cholesterol, HDL and LDL) were analyzed using the Alere Cholestech LDX point of care analyzer.

*Dietary intake* was measured via two 24-h dietary recalls conducted at each assessment time point using the Automated Self-Administered 24-Hour Dietary Recall (ASA-24) from the National Cancer Institute [26]. Participants were provided with pre-established logins and prompted to log in to the ASA-24 website at an unscheduled time and complete a 24-h recall on one weekday and one weekend day, at which time they reported everything they had to eat and drink in the 24 h on the day prior.

*Physical activity* was measured using a staff-administered version of the Paffenbarger Physical Activity Questionnaire (PAQ) at each assessment period [27]. Using a guided-interview format, this measure assesses leisure-time physical activity, walking, and stair-climbing over the previous week. Although originally developed as a self-report measure, the interview format allows for clarification of the activities and activity intensity unavailable when using the questionnaire alone.

Participants also completed a measure of quality of life at baseline and 4 months using the CDC Health-Related Quality of Life scale [28]. This is a 4-item measure that assesses perceptions of general health, including self-reported ratings of number of unhealthy physical and mental health days in the last 30 days. In this study the measure had acceptable internal consistency (α = .70). The Physical Activity Group Environment Questionnaire (PAGEQ) is a 26-item measure that assessed group cohesion at 4 months (α = .96) [29]. The measure assessed individuals’ perceptions of the cohesiveness of the group sessions and was modified for this study to assess a weight loss group. Program adherence was measured by recording attendance at face-to-face group sessions and logins to the study website. Participants also completed a study-developed program satisfaction questionnaire at 4 months.

### Statistical methods

The primary outcome of change in weight, as well as secondary outcomes of percent weight loss, change in blood pressure, cholesterol, HbA1c, general health, calorie intake, and calorie expenditure, were analyzed using intent-to-treat methodology. Multiple imputation using Markov chain Monte Carlo (MCMC) method was used to replace missing values. PROC MI and PROC MIANALYZE in SAS 9.3 were used to conduct repeated-measures mixed-model analysis with age and race as covariates to estimate primary and secondary outcomes and to test group differences in change over time [9, 30]. Race and age were included as covariates in our analysis as they have been shown in prior studies to be associated with weight loss [31, 32]. Individuals who reported using medication for hypertension were censored for analyses of change in blood pressure. Similar methodology was used for analyses of total cholesterol and HbA1c. This program was designed to be inclusive (i.e., did not include an upper BMI cut off) and included a participant with a weight that was a statistical outlier located six standard deviations above the mean. Analyses were conducted both with and without this case (data not shown) and were similar. The results presented follow intention to treat and include the outlying case.

Given the pilot nature of this study, analyses presented here were conducted using the individual as the unit of analysis. Sensitivity analyses were also conducted considering the social grouping of participants with their friend and family. These were unadjusted analyses that used the average weight loss of the social group (containing between 1 and 4 individuals) as the unit of analysis with baseline observations carried forward for any missing data at 4 months. Analyses conducted considering the clustering of participants yielded results for weight and secondary outcomes that were similar to those when using the individual as the unit of analysis. Therefore, we subsequently present the results of the sensitivity analyses for the primary outcome of weight in the text, but not for the remaining outcomes.

Program retention, adherence, and satisfaction measures were compared using t-tests and chi-squared tests. Group comparisons with skewed distributions were analyzed using Wilcoxon-Mann-Whitney tests and are reported with medians and interquartile ranges. Relationships between program participation and weight loss were assessed with Spearman correlations. Finally, logistic regressions were used to test baseline characteristics as predictors of achieving a five-percent weight loss.

## Results

**Fig. 1:**
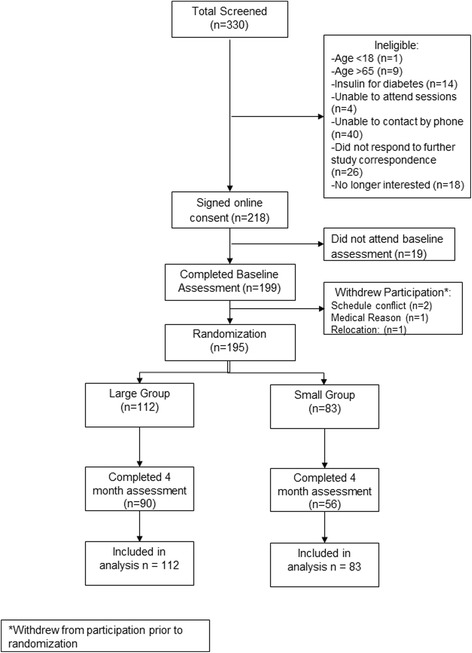
CONSORT Diagram

Table 1 describes the baseline characteristics of participants in the Lose Now NC program (*N* = 195). Participant flow is shown on Fig. 1 [33]. Overall, 75% of participants returned for the 4 month follow-up visit. Participants in the LG were more likely to return for the 4-month assessment visit than those in the SG (80.4% vs. 67.5%, χ^2^ = 4.21, *p* = 0.04). Individuals who reported taking medications for hypertension, hyperlipidemia, or oral medications for diabetes were more likely to complete the 4-month assessment than those not reporting medication use at baseline (82.9% vs. 69.0%, χ^2^ = 4.88, *p* = 0.03). There were no other differences on baseline characteristics between those who completed the study and those who did not (all *p*’s > 0.17).

**Table 1:** Baseline characteristics by treatment group

	Total Sample (*N* = 195)	Large Group (*N* = 112)	Small Group (*N* = 83)
Age (y) [m ± sd]	46.3 ± 10.8	45.0 ± 10.4	48.2 ± 11.1
Female [n (%)]	176 (90.3%)	102 (91.1)	74 (89.2)
Education^1^			
High school or less	19 (9.8)	12 (10.8)	7 (8.4)
Some college	80 (41.2)	39 (35.1)	41 (49.4)
College graduate or beyond	95 (49.0)	60 (54.1)	35 (42.2)
Race or ethnic group			
White	88 (45.1)	58 (51.8)	30 (36.1)
Black	101 (51.8)	52 (46.4)	49 (59.0)
Other	6 (3.1)	2 (1.8)	4 (4.8)
Married or living with partner	124 (63.6)	70 (62.5)	54 (65.1)
Current smoker	15 (7.7)	7 (6.3)	8 (9.6)
Medications^2^	82 (42.1)	44 (39.3)	38 (45.8)
Weight (kg)	103.7 ± 27.1	102.7 ± 23.7	105.0 ± 31.3
BMI (kg/m^2^)	37.9 ± 8.4	37.6 ± 8.0	38.3 ± 8.9
Systolic blood pressure (mm Hg)	127.4 ± 14.7	126.5 ± 14.6	128.6 ± 15.0
Diastolic blood pressure (mm Hg)	75.1 ± 10.7	74.0 ± 11.1	76.5 ± 9.9
Total cholesterol (mg/dL)	188.0 ± 35.1	186.0 ± 34.8	190.8 ± 35.7
HbA1c	5.6 ± 0.6	5.6 ± 0.7	5.7 ± 0.6
Enrolled with friends/family	134 (68.7)	75 (67.0)	59 (71.1)

Notes. ^1^n = 194; ^2^Medications for hypertension, hyperlipidemia, or oral medications for diabetes

### Intervention adherence

**Fig. 2:**
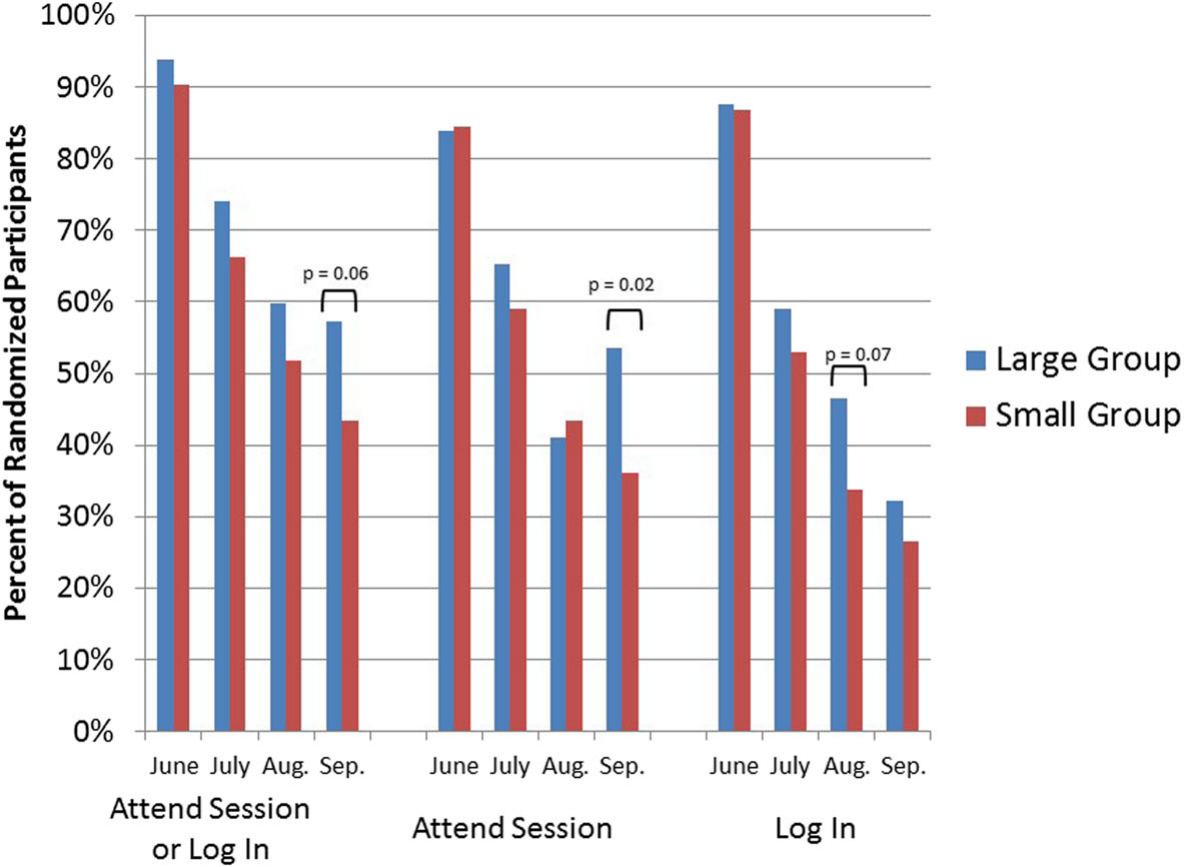
Session attendance and online program use by treatment group and program month

Attendance at the 4 monthly treatment group meetings did not differ by group. LG and SG participants did not differ in the number of sessions attended (median, interquartile range (IQR); LG: 3, 1–4; SG: 2, 1–4; z = −1.00, *p* = 0.32) nor number of times they logged into the study website (LG: 22, 3.5–56.5; SG: 9, 2–47, z = −1.44, *p* = 0.15). Figure 2 shows the percentage of participants who attended each group session, logged into the website during each month of the program, and the percentage who either attended the session or logged into the website. The only significant difference between groups was that a greater percentage of participants in LG attended the final group session compared to SG (χ^2^ = 5.83, *p* = 0.02). Thirteen participants (6 LG, 7 SG) participants never logged into the website and attended no sessions; there was no difference between groups (χ^2^ = 0.73, *p* = 0.39). Participants monitored their dietary intake on the website on average 26.4 days (SD = 31.5), with no difference between treatment groups. More participants used the web diary (*n* = 73) than tracking on their own and manually entering their total calories (*n* = 19), while 61 participants used a combination of both. Forty-two participants did not use either form of monitoring. There were no differences in type of monitoring used between groups.

### Weight loss and secondary outcomes

As shown in Table 2, both groups in the study experienced significant weight losses (LG: −4.1 kg, 95% CI: -5.1, −3.0; SG: -3.7 kg, 95% CI: -5.0, −2.4) at the 4-month assessment, however, there was no difference between groups (*p* = 0.67). Sensitivity analyses using average weight loss of each social group as the unit of analysis had similar results, with no difference between groups (*p* = .20). There were no differences in completion of the follow-up assessment by those who enrolled with friends and family (71.2%) or alone (65.3%; *p* = 0.43). Among those who completed the assessment, there were no differences in weight loss based on enrollment status (friends/family: −3.7 kg ± 5.2; alone −4.2 kg ± 4.2; *p* = 0.56).) A similar proportion of participants in each treatment group who returned for the follow-up visit lost at least 5% of their initial body weight during the program (LG: 35.6%, SG: 28.6%, χ^2^ = 0.76, *p* = 0.38). Weight loss was associated with sessions attended (Spearman’s rho = −0.25, *p* = 0.002), and logins to the study website (rho = −0.50, *p* < 0.001). Participants who lost at least 5% of their initial body weight were more likely to be white (OR = 2.28; 95% CI: 1.17, 4.43) and older (OR = 1.03; 95% CI 1.00, 1.07) than participants who did not reach this goal. No other baseline characteristics were associated with achieving a 5% weight loss.

**Table 2:** Anthropometric, diet, physical activity, and cardiometabolic changes^a^

			*p*-values		
	Baseline^b^	Change at 4-months	Time	Group	Time x Group
Weight (kg)			**<0.001**	0.50	0.67
Large group	102.5 (97.6, 107.5)	−4.1 (−5.1, −3.0)			
Small group	105.2 (99.5, 111.0)	−3.7 (−5.0, −2.4)			
Weight (%)			**<0.001**	0.72	0.52
Large group	0	−4.0 (−5.0, −3.0)			
Small group	0	−3.5 (−4.7, −2.3)			
Systolic Blood Pressure (mm Hg)			**<0.001**	0.63	0.77
Large group	123.9 (120.7, 127.1)	−5.9 (−8.8, −3.0)			
Small group	125.2 (121.2, 129.1)	−5.1 (−10.8, 0.7)			
Diastolic Blood Pressure (mm Hg)			0.25	**0.02**	0.56
Large group	72.0 (69.6, 74.3)	−1.5 (−4.0, 1.0)			
Small group	76.5 (73.6, 79.4)	0.02 (−4.9, 5.0)			
Total Cholesterol (mg/dL)			0.15	0.07	0.29
Large group	183.6 (177.2, 189.9)	−3.3 (−7.9, 1.2)			
Small group	192.8 (185.0, 200.6)	−7.9 (−15.3, −0.6)			
HbA1c ^e^			**<0.001**	0.16	0.18
Large group	5.5 (5.4, 5.6)	−0.1 (−0.2, −0.1)			
Small group	5.6 (5.5, 5.7)	−0.2 (−0.2, −0.1)			
General health rating			**0.01**	0.94	0.95
Large group	2.9 (2.7, 3.0)	−0.2 (−0.4, −0.04)			
Small group	2.9 (2.7, 3.0)	−0.2 (−0.4, −0.00)			
Caloric Intake (kcal)			**<0.001**	0.14	0.69
Large group	1985 (1866, 2105)	−316 (−492, −139)			
Small group	1844 (1705, 1984)	−378 (−621, −136)			
Caloric Expenditure (kcal)^c^			**0.005**	0.79	0.67
Large group	278 (194, 780)	254			
Small group	299 (197, 456)	191			

^a^Intention-to-treat with multiple imputation repeated-measures mixed-model analysis with age and race as covariates. n = 112 for LG and n = 83 for SG for all analyses except those for BP, total cholesterol and HbA1c. Adjustments were made to the sample to exclude for BP, lipid lowering, and diabetes medication use. For BP: *n* = 71 for LG and *n* = 47 for SG. For total cholesterol: *n* = 97 for LG and *n* = 65 for SG. For HbA1c: *n* = 101 for LG and *n* = 80 for SG. Statistically significant *P* values are shown in bold

^b^All values are model predicted means; 95% CIs in parentheses

^c^Analyses conducted using log-transformed values

There were improvements in systolic BP, HbA1c, and self-reported health ratings over time (*p*’s < 0.01); however, there were no differences by group (*p*’s > 0.18). There were no significant changes to total cholesterol or diastolic BP over time (*p*’s > .15). Participants in both groups reported decreased caloric intake at 4 months and increased caloric expenditure in leisure time physical activity (*p*’s < 0.005), with no difference in the magnitude of the dietary and activity changes made by treatment group.

Among those who completed the 4-month assessment, weight loss was associated with change in calorie intake (rho = 0.26, *p* = 0.004), self-reported ratings of general health (rho = 0.18, *p* = 0.04), and change in HbA1c (rho = 0.38, *p* < 0.001). Weight loss was not associated with change in self-reported physical activity (rho = −0.15, *p* = 0.06), change in blood pressure (systolic, rho = 0.18, *p* = 0.10; diastolic rho = 0.05, *p* = 0.66) or change in cholesterol (rho = 0.08, *p* = 0.36).

### Satisfaction with program

Seventy-three percent (142 out of 195) of participants completed questions focused on program satisfaction. Mirroring the overall completion rate of the assessment visit, somewhat more participants in the LG completed the survey than the SG (78.6% vs. 65.1%, χ^2^ = 4.40, p = 0.06). Among those who completed the survey, 94.4% reported they were “satisfied” or “very satisfied” with the program they received (no difference between groups χ^2^ = 0.61, *p* = 0.43). Nearly all participants reported that they would recommend the program to a friend; however, this was slightly lower among participants in the SG than in the LG (92.6% vs. 98.9%, χ^2^ = 3.82, *p* = 0.05). In ratings of individual components of the face-to-face and online program, the groups reported similar levels of satisfaction with the group meetings (97.5% satisfaction), weigh-in process (98.4%), and session topics (96.7%). These measures did not differ between groups (*p*’s > 0.37). As shown in Table 3, group cohesion measures indicated that the LG and SG participants reported similar attraction to the group they were assigned to and felt similar levels of integration with the group members (all *p*’s > 0.19).

**Table 3:** Group Cohesion at 4 months^a^

	Large Group	Small Group	*p*-value
N	88	54	
Attraction to group-Task^b^	7.0 ± 1.2	6.8 ± 1.4	0.31
Attraction to group- Social	5.6 ± 1.9	6.0 ± 1.6	0.21
Integration with group- Task	6.3 ± 1.2	6.1 ± 1.5	0.41
Integration with group- Social	5.4 ± 1.3	5.1 ± 1.4	0.19
Attraction to group leader	6.1 ± 1.5	6.6 ± 1.6	0.08

^a^Difference between means tested using independent t-tests. Values are mean ± standard deviation

^b^Subscale scores range from 1 to 9 with higher scores indicating greater attraction/integration

## Discussion

The objective of the present study was to compare the effects of treatment group size in the context of a hybrid program using the Internet and monthly face-to-face groups. The study delivered treatment using an Internet program coupled with either monthly large group (100 persons) or smaller groups (20 persons). Participants in both large and small treatment group formats showed significant weight loss over the 4 month intervention (approximately 4 kg) and weight losses did not differ significantly between groups. Weight losses were approximately 4% at 4 months and were associated with reduction in HbA1c.

This study is among the first to investigate delivering behavioral weight loss in a group of more than 100 individuals and to examine group size in the context of supplementing a primarily Internet delivered program. Though other studies examining group size used large groups of a similar size to the “small” groups used here (approximately 20–30), our results are similar to the Look Ahead findings where group size was not related to weight loss [10]. In contrast, Dutton et al. [5] found weight losses to be 3–4 kg better in smaller groups (8–10 participants) compared to larger groups (30 participants), and this was partially explained by adherence such that small group participants showed better self-monitoring and a trend toward better session attendance. Participants in the current study were encouraged to join the study with friends or family members, so it is possible that participating in a large group was less overwhelming with existing social support members than participating alone. Moreover, because much of the treatment was actually delivered online, it may be that more than four in-person treatment sessions are needed to see an effect of group size, as found in the Dutton study with 24 weekly sessions. Finally, it may be necessary to use much smaller groups of approximately 10 individuals to see the benefits of small group size.

We have previously reported on a Stepped Care model for delivering behavioral weight loss that began with monthly group contact (groups of approximately 25) and utilized postal mail for lesson and diary exchange between monthly sessions [20]. At 3 months, weight losses averaged 5.9% in the group receiving monthly group treatment, somewhat better than reported here; however participants in that study had to agree to be randomized to receive either weekly or monthly group treatment sessions which may reflect a different study sample. The weight losses achieved in this study were similar to those in Leahey et al. [19]. The Leahey study reported average weight losses of approximately 5% after 4 months using a hybrid in-person and online approach, however the in-person meetings were more frequent (weekly versus monthly) and were optional. Interestingly, the hybrid approach produced better maintenance in that study; our study did not have a longer term follow-up visit. Though few studies have examined ways to augment remotely delivered treatments with in-person sessions, future research might examine the size, frequency, and timing of face-to-face interaction that produce the best outcomes.

Though not statistically significant, it is worth noting that the direction of the observed means in this study were in a direction favoring the LG (weight loss, attendance, logins etc.). LG participants attended 25% more treatment sessions compared with small group participants (3 vs. 2). They also logged into the study website twice as many times over the 4 months (22 vs. 9 times), on average. Retention was significantly greater in the LG compared with SG (*p* < .05). When we designed the study we were unsure whether the large group might lead to greater feelings of anonymity, and if so, whether this would be beneficial or detrimental for attendance. Our measure of group cohesion did not show differences on feelings of integration with group members, and while program satisfaction was high in both groups, 99% of LG participants would recommend the program to a friend. Taken together, these metrics suggest that engagement and satisfaction was not worse, and may have been somewhat better in the LG participants compared with the SG.

To control for potential effects of treatment group leaders, all four SG treatment leaders participated as leaders in various sessions in the LG. However, to encourage participation with the large number of people necessitated conducting the LG in a somewhat different manner than the SG. For example, text message/internet polling were used during the sessions to allow participants to express how they had done the previous week, amount of weight lost, etc. and view group level feedback instantaneously. Similarly, participants could text in questions or problems at any time and the treatment providers address questions during the session. These aspects may have created a fun and engaging atmosphere. Future research might examine approaches to interaction in groups in addition to group size.

This study was not designed to study non-inferiority, however, future well powered studies might include careful cost tracking to determine the cost-effectiveness of behavioral weight loss delivered in substantially larger groups. Similarly, we employed a novel method of recruiting in a community setting - encouraging enrollment with family or friends. This study was also not designed to examine the effects of enrolling with others on weight loss but this method of delivering treatment in a large group may be of interest to those seeking to study social influences on weight loss.

One of the notable strengths of this study is the diversity represented in the sample. Prior to recruitment for the randomized intervention trial, we conducted focus groups of men (any race) and African American women to gather feedback on recruitment messages and message placement that would maximize recruitment of groups that are typically underrepresented in behavioral weight loss trials. Consensus from participants was that joining a weight loss program as part of a research study would be highly favorable and most were likely to attend the monthly visits and use the web-based program, suggesting feasibility of our approach. Sample recruitment flyers with images, taglines and a brief description of the program were also presented to the groups. The results of the focus groups were summarized in a report and provided to a marketing company for finalization of the study recruitment website and advertisements. Our recruitment approach also utilized a community member who was a Black male. These combined approaches resulted in a yield of 50% of the participants self-identifying as African American and fewer than 50% reporting a college degree which lends to the generalizability of the findings. Despite these efforts, men were underrepresented in our sample, indicating a need for further study of how men can be recruited to participate in weight loss programs [34]. A further strength of this study was that weight was measured objectively by blinded assessors. The weight losses were modest, 3–4%, however the study was 4 rather than 6 months duration, as is commonly reported in the literature and used tailored, automated feedback rather than human email counseling; thus the human counseling support was limited to the monthly face-to-face group sessions. The study is also limited by the short duration, modest follow-up rates (75%), self-report measures of diet and activity, and our ability to study only a single large group. Furthermore, we are unable to separate group size from other aspects of treatment delivery as noted above. Future trials should control for this.

## Conclusions

The results of this study suggest that using a hybrid approach of in-person and online weight loss interventions may be an effective way to reach larger and more diverse populations. While a fully-powered study with multiple large groups is needed to confirm this initial finding, this study provides preliminary evidence that moving beyond modest-sized treatment groups (20 people) may be a worthwhile direction for increasing the cost-effectiveness of group-based behavioral weight loss interventions. Further, it will be important for future studies to investigate the mechanisms through which larger versus smaller groups operate and perhaps whether smaller versus larger groups may be more appropriate for use with mobile technologies, specific demographic subgroups, or phases of treatment.
